# Early Detection and Monitoring of Nephrolithiasis: The Potential of Electrochemical Sensors

**DOI:** 10.3390/s25082547

**Published:** 2025-04-17

**Authors:** Kaiqiang Sun, Ningbin Zhao, Peizheng Shi, Zhuang Sun, Chen Ye, Li Fu, Dan Dai, Wubo Chu, Tao Cai, Hsu-Sheng Tsai, Cheng-Te Lin

**Affiliations:** 1 School of Materials Science and Chemical Engineering, Ningbo University, Ningbo 315211, China; sunkaiqiang@nimte.ac.cn; 2 Qianwan Institute, Ningbo Institute of Materials Technology and Engineering (NlMTE), Chinese Academy of Sciences, Ningbo 315201, China; zhaoningbin@nimte.ac.cn (N.Z.); shipeizheng@nimte.ac.cn (P.S.); sunzhuang@nimte.ac.cn (Z.S.); yechen@nimte.ac.cn (C.Y.); daidan@nimte.ac.cn (D.D.); chuwubo@nimte.ac.cn (W.C.); caitao@nimte.ac.cn (T.C.); 3 State Key Laboratory of Advanced Marine Materials, Ningbo Institute of Materials Technology and Engineering (NIMTE), Chinese Academy of Sciences, Ningbo 315201, China; 4 Center of Materials Science and Optoelectronics Engineering, University of Chinese Academy of Sciences, Beijing 100049, China; 5 College of Materials and Environmental Engineering, Hangzhou Dianzi University, Hangzhou 310018, China; fuli@hdu.edu.cn; 6 College of Chemical Engineering, Zhejiang University of Technology, Hangzhou 310014, China; 7 Laboratory for Space Environment and Physical Sciences, Harbin Institute of Technology, Harbin 150001, China; hstsai@hit.edu.cn; 8 School of Physics, Harbin Institute of Technology, Harbin 150001, China

**Keywords:** nephrolithiasis, electrochemical sensors, oxalate, uric acid, point-of-care testing

## Abstract

Nephrolithiasis (kidney stone disease) continues to pose a significant global health challenge, affecting millions of individuals and placing substantial economic pressures on healthcare systems. Traditional diagnostic methods—such as computed tomography (CT), ultrasound, and basic urinalysis—are often limited by issues including radiation exposure, lower sensitivity in detecting small stones, operator dependency, and the inability to provide real-time analysis. In response, electrochemical sensors have emerged as innovative and powerful tools capable of the rapid, sensitive, and specific detection of key biomarkers associated with nephrolithiasis. This review highlights the advances in electrochemical approaches for monitoring oxalate and uric acid, the two primary metabolites implicated in kidney stone formation. We discuss the principles of electrode design and fabrication, including nanomaterial integration, 3D printing, and molecular imprinting, which have markedly improved detection limits and selectivity. Furthermore, we critically evaluate the practical challenges—such as sensor fouling, reproducibility, and stability in complex biological matrices—that currently impede widespread clinical implementation. The potentials for miniaturization and point-of-care integration are emphasized, with an eye toward continuous or home-based monitoring systems that can offer personalized insights into risk of stone formation and progression. By consolidating recent findings and exploring future trends in multi-analyte detection and wearable diagnostics, this review provides a roadmap for translating electrochemical sensors from research laboratories to routine clinical practice, ultimately aiming to enhance early intervention and improve patient outcomes in nephrolithiasis.

## 1. Introduction

Nephrolithiasis [1,2], commonly known as kidney stone disease, has emerged as a significant global health concern with far-reaching implications for patients’ quality of life and healthcare systems worldwide. The prevalence of this condition has shown a marked increase over recent decades [3], warranting increased attention from the medical community and researchers alike. The prevalence of kidney stones among U.S. adults has remained relatively stable, with a prevalence rate of approximately 9.9% from 2017 to 2020 [4]. Among those with kidney stones, 54.8% were male, and 45.2%were female (*p*-value < 0.05) [5]. The impacts of kidney stones on patients’ quality of life are profound and multifaceted [6]. Individuals suffering from nephrolithiasis often experience severe pain, commonly described as one of the most intense forms of pain imaginable [7]. This pain, often accompanied by nausea, vomiting, and hematuria, can be debilitating and significantly disrupt daily activities [8]. A study was conducted using the Wisconsin stone quality of life questionnaire [9] revealed that patients with kidney stones reported lower scores across all the domains of the SF-36 health-related quality-of-life questionnaire compared to the general population. Particularly notable were reductions in physical functioning, bodily pain, and general health perceptions, underscoring the pervasive impact of this condition on overall wellbeing [10]. Beyond the immediate physical discomfort, nephrolithiasis poses substantial economic burdens on both individual patients and healthcare systems [11]. In the United States alone, the annual cost associated with kidney stone disease was estimated to be $10 billion in 2021 [12], a figure that has likely increased since then because of the rising prevalence and healthcare costs. This economic impact is compounded by lost productivity, with patients often requiring time off work for treatment and recovery. A survey counted all the hospitalized patients with kidney stones coded as a primary diagnosis or complication in Spain during the period 2017–2020, with a mean length of stay of 5.23 days (95% CI: 5.06–5.39) [13], translating to significant economic losses at both personal and societal levels.

The global epidemiology of kidney stones presents a complex picture [14,15,16,17], Historically, kidney stone disease was more prevalent in men than in women, with a ratio of approximately 3:1 [18]. However, recent data suggest this gap is narrowing, with some studies reporting ratios closer to 1.3:1 [19]. This shift is particularly pronounced in younger age groups, indicating changing risk factors that may be disproportionately affecting women. Geographically, the highest prevalence rates are observed in countries with hot, arid climates, such as those in the Middle East, where rates can exceed 20% in some populations [20]. However, increasing rates are being observed globally, including in regions previously considered as low-risk, suggesting a complex interplay of genetic [21,22], dietary [23,24], and environmental factors [25]. The clinical importance of nephrolithiasis extends beyond its immediate symptoms and quality-of-life impacts [26]. Kidney stones are associated with an increased risk of chronic kidney disease, with a study by Zisman et al. [27] demonstrating that stone formers had a 50–67% higher risk of developing chronic kidney disease compared to non-stone formers. Furthermore, recent research [28] has uncovered associations between kidney stone disease and other systemic conditions, including cardiovascular disease and metabolic syndrome, highlighting the potential for nephrolithiasis to serve as a marker for broader health risks. Given the significant personal, societal, and economic impacts of kidney stone disease, there is a pressing need for improved diagnostic and management strategies. This need forms the primary motivation for ongoing research in this field, with the ultimate goal of reducing the burden of nephrolithiasis through early detection, accurate diagnosis, and effective prevention strategies. The development of novel diagnostic approaches holds the potential to revolutionize the management of kidney stone disease, offering opportunities for earlier intervention and more personalized treatment strategies.

**Figure 1:**
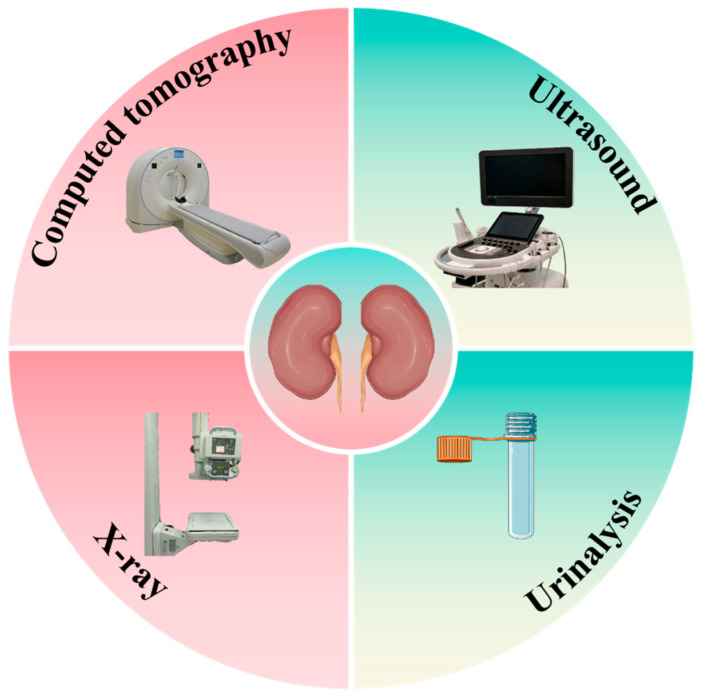
Common techniques for detecting kidney stones.

Currently, the diagnosis of kidney stones relies predominantly on imaging techniques, such as computed tomography (CT) [29], ultrasound [29], and X-ray [30], complemented by urinalysis [31] (Figure 1). Although these methods have been the mainstay of nephrolithiasis diagnosis for decades, they are not without limitations. CT scans, although highly sensitive and specific for detecting kidney stones, expose patients to ionizing radiation, limiting their use in repeated assessments and in certain patient populations, such as pregnant women. Ultrasound [32], although safer, has lower sensitivity, particularly for smaller stones, and its accuracy is highly operator dependent. Traditional urinalysis, although useful for detecting associated urinary abnormalities, lacks the specificity to definitively diagnose or characterize kidney stones. Moreover, these conventional methods often fall short of providing real-time, point-of-care diagnostics that could facilitate rapid clinical decision making. They also offer limited [33] information about the stone composition and formation process, crucial factors in determining appropriate treatment and prevention strategies. The need for specialized equipment and trained personnel further limits the accessibility of these diagnostic tools, particularly in resource-limited settings.

In light of these limitations, there is growing interest in the potential of electrochemical sensors for nephrolithiasis diagnosis. Electrochemical methods [34,35] offer several distinct advantages [36,37,38] that position them as promising candidates for next-generation diagnostic tools in kidney stone management. First, electrochemical sensors [39,40,41] have the potential to provide rapid [42,43,44], real-time [45] results, enabling point-of-care testing that could drastically reduce diagnostic delays. This speed of the analysis could be particularly valuable in emergency settings, where rapid diagnosis is crucial for effective pain management and treatment planning [46]. Second, electrochemical sensors can be designed to detect specific molecular markers associated with kidney stone formation [47], offering a level of specificity that traditional urinalysis cannot match. This capability could enable not only the detection of kidney stones but also provide insights into their composition and the underlying metabolic abnormalities contributing to their formation. Such information is invaluable for tailoring preventive strategies and guiding treatment decisions. Third, the potentials for miniaturization [48] and integration [49] with portable devices make electrochemical sensors highly promising for developing accessible, user-friendly [50] diagnostic tools. This could expand the reach of kidney stone diagnostics beyond hospital settings, potentially enabling home monitoring for high-risk individuals or follow-up testing in primary care settings. Furthermore, electrochemical sensors offer the possibility of continuous or frequent monitoring [51], which could be particularly valuable in tracking the progression of stone formation or the effectiveness of preventive measures over time. This capability aligns well with the growing emphasis on personalized medicine and proactive health management.

In this review, we first provide an overview of kidney stone disease, emphasizing its rising incidence, associated socioeconomic burden, and the drawbacks of traditional diagnostic methods. Recognizing the urgency for improved approaches, we then elucidate the fundamentals of kidney stone formation and identify the primary biomarkers—namely, oxalate and uric acid—which accurate, real-time detection is vital for better prevention and management. In contrast, there is still a lack of consensus on the concentration thresholds and assay specificity of other biomarkers, including hypoxanthine, proline, aspartate, neutrophil-gelatinase-associated lipofuscin, and renal injury molecules, in stone formation, which are currently more applicable to mechanistic studies at the scientific level than to clinical warnings. Subsequently, we delve into the design, fabrication, and operational principles of electrochemical sensors, highlighting various sensing modalities and their capacities to offer enhanced sensitivity, selectivity, and portability compared to those of conventional techniques. By examining recent advancements and comparative studies, this review underscores the pivotal roles of novel electrode materials, nanotechnology integration, and molecular imprinting in lowering detection limits and bolstering sensor stability. Furthermore, we present a critical appraisal of practical applications, covering benchtop analyses, point-of-care diagnostics, and prospective wearable or remote-monitoring systems. Our motivation for writing this review lies in bridging the gap between emerging sensor technologies and unmet clinical needs, as early kidney stone detection could substantially reduce patient suffering and healthcare costs. By examining both the promise and the challenges of electrochemical approaches, we aim to provide researchers and clinicians with a comprehensive resource for future development. Ultimately, this review seeks to catalyze interdisciplinary efforts that will facilitate the translation of these innovative sensors into routine clinical practice, thereby transforming the landscape of nephrolithiasis diagnosis and patient care.

## 2. Mechanisms of Kidney Stone Formation and Associated Biomarkers

### 2.1. Stone Compositional Analysis

**Figure 2:**
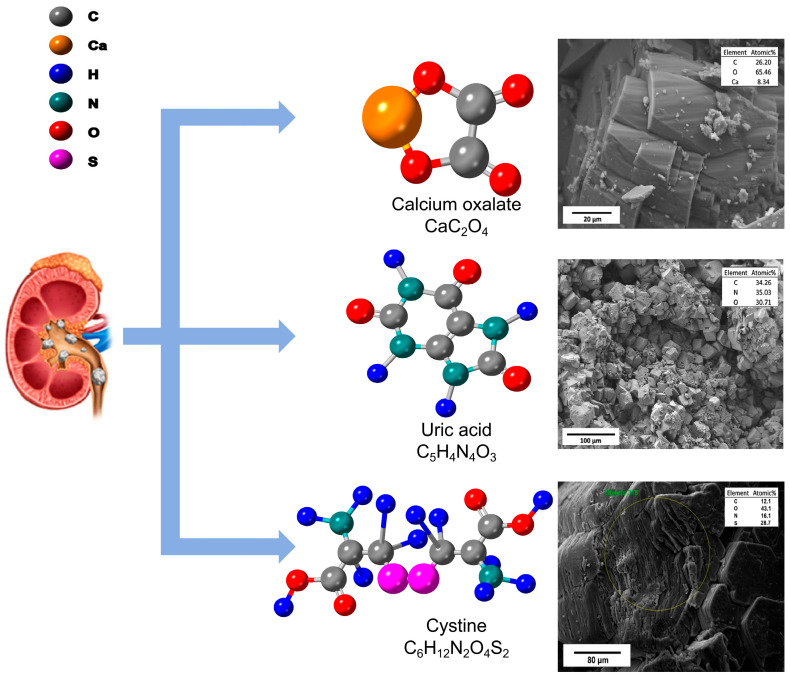
The chemical structures of the three types of stones, as well as their images from top to bottom are calcium oxalate [72], uric acid [72], and cystine stones [73]. Reproduced with permission.

Kidney stones, also known as renal calculi, are solid masses that form in the urinary tract [52,53]. These stones can vary significantly in their chemical composition [54,55,56], with the most common types being calcium oxalate, uric acid, and cystine stones (Figure 2). Understanding the chemical structure of each stone type is crucial for effective diagnosis and treatment.

Calcium oxalate [57] stones are the most prevalent type, accounting for approximately 80% of all kidney stones [58]. These stones are composed of calcium ions bound to oxalate ions. The chemical structure of calcium oxalate can exist in two primary forms: calcium oxalate monohydrate (COM) [59] and calcium oxalate dihydrate (COD) [60]. COM, also known as whewellite, is the more stable and common form, characterized by its monoclinic crystal structure. COD, or weddellite, has a tetragonal crystal structure and is less stable, often transforming to COM over time [61].

Uric acid stones are the second most common type, comprising about 5–10% of all kidney stones [62,63,64]. These stones form when there is an excess of uric acid in the urine, often because of increased purine metabolism or decreased uric acid excretion [65]. Uric acid stones have a chemical formula of C_5_H_4_N_4_O_3_ and typically form rhombic or needle-like crystals. Unlike calcium oxalate stones, uric acid stones are radiolucent, making them difficult to detect in standard X-ray images [66].

Cystine stones are relatively rare, accounting for approximately 1–2% [67] of all kidney stones. They develop primarily because of cystinuria [68], a hereditary metabolic disorder characterized by the impaired renal tubular reabsorption of dibasic amino acids—including cystine, ornithine, lysine, and arginine. As a result, high urinary cystine concentrations lead to precipitation, forming crystals that often exhibit a characteristic hexagonal shape [69]. Chemically, cystine [70] is formed by the disulfide linkage of two cysteine molecules (HS–CH_2_–CH(NH_2_)–COOH), making it poorly soluble in urine. Early identification of cystinuria is crucial, as cystine stones tend to recur and can be challenging to treat [71]. Standard imaging techniques, along with appropriate biochemical tests, are typically employed for their detection and management.

### 2.2. Roles of Biomarkers in Kidney Stone Formation

#### 2.2.1. Oxalate

**Figure 3:**
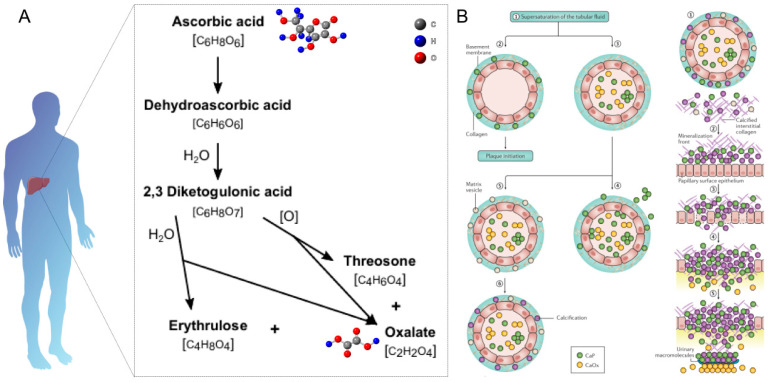
(**A**) Schematic diagram of the metabolism of ascorbic acid to oxalate in humans [76]. (**B**) Schematic diagram of the mechanism of oxalate stone formation [53].

Calcium oxalate stones are the most common pathological mineralization product of the urinary system, and their formation mechanism is closely related to oxalate metabolism disorders [74]. In order to elucidate the molecular basis of stone formation and to develop targeted intervention strategies, a detailed understanding of the oxalate metabolic regulatory network is important. As shown in Figure 3A, the metabolic pathway of oxalate is complex and involves both endogenous production and exogenous sources. In the body, oxalate is primarily produced as an end product of vitamin C metabolism in the liver [75,76]. The enzyme lactate dehydrogenase catalyzes the conversion from glyoxylate to oxalate, while another pathway involves the oxidation of glycolate by glycolate oxidase [77,78]. Several factors influence urinary oxalate levels, including diet, gut absorption, and endogenous production. Dietary sources of oxalate include spinach, rhubarb, nuts, and chocolate [79,80]. The absorption of dietary oxalate occurs primarily in the colon, where it can be influenced by the presence of calcium, which can bind to oxalate and reduce its absorption. Certain gastrointestinal disorders, such as inflammatory bowel disease or short bowel syndrome, can increase oxalate absorption, leading to hyperoxaluria [81].

The mechanism of calcium oxalate stone formation involves a complex interplay of supersaturation, crystal nucleation, and growth (Figure 3B) [82,83]. When the concentrations of calcium and oxalate in the urine exceed their solubility product, the urine becomes supersaturated. This supersaturation leads to the formation of crystal nuclei, which serve as the foundation for stone growth. As more calcium and oxalate ions attach to these nuclei, the crystals grow larger and may aggregate, eventually forming a kidney stone. Factors such as the urinary pH, the presence of promoters (e.g., uric acid) or inhibitors (e.g., citrate), and urinary flow rate can all influence this process.

#### 2.2.2. Uric Acid

**Figure 4:**
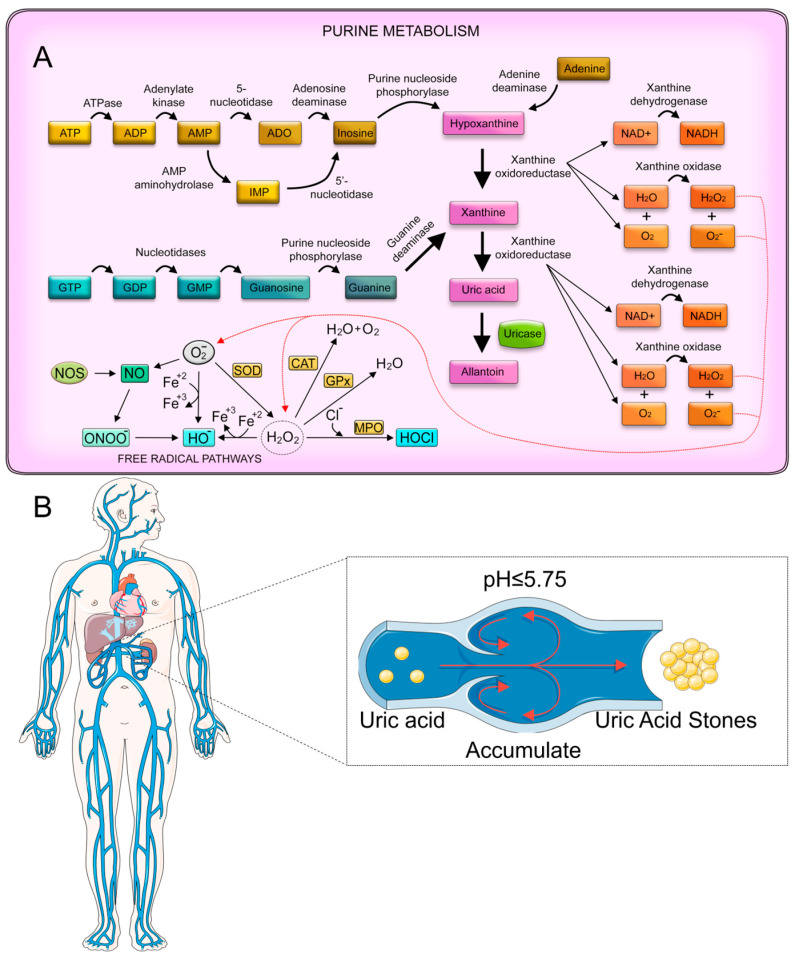
(**A**) Purine metabolism and oxidative stress [85]. Copyright 2021, from Elsevier. (**B**) The formation of uric acid stones.

Abnormalities in uric acid metabolism not only lead directly to the formation of uric acid stones but also promote the heterogeneous nucleation and growth of calcium oxalate stones through a variety of mechanisms [84]. This synergistic pathogenic effect is receiving increasing clinical attention. The metabolic pathway of uric acid is closely linked to purine metabolism (Figure 4A). Purines, which are components of DNA and RNA, are broken down into xanthine, which is then oxidized by xanthine oxidase to form uric acid [85]. In humans, uric acid is the final product of purine metabolism, as we lack the enzyme uricase, which further breaks down uric acid in most other mammals [86]. Urinary uric acid levels are influenced by various factors [87], including diet, purine metabolism, and certain medical conditions. A diet high in purine-rich foods, such as red meats, organ meats, and some seafoods, can increase uric acid production. Metabolic disorders, like gout, or conditions that cause rapid cell turnover (e.g., certain cancers or chemotherapy) can also lead to elevated uric acid levels [88]. Additionally, certain medications and genetic factors can affect uric acid excretion by the kidneys [84].

The mechanism of uric acid stone formation is primarily driven by the urinary pH level (Figure 4B). Uric acid is a weak acid, with a p*K*_a_ of 5.75, meaning it exists predominantly in its undissociated form at pH levels below 5.75. In an acidic urine environment, uric acid becomes less soluble, leading to crystal formation. As the urine pH drops, the solubility of the uric acid decreases drastically, promoting stone formation. Other factors, such as low urine volume and increased uric acid excretion, can exacerbate this process [89].

#### 2.2.3. Medical Detection of Relevant Metabolic Indicators

The accurate measurement of urinary biomarkers is essential for the diagnosis, treatment, and prevention of kidney stones. Current clinical methods for measuring these biomarkers involve a combination of urine and blood tests, each with its own strengths and limitations.

Oxalate measurement typically involves 24 h urine collection, followed by analysis using high-performance liquid chromatography (HPLC) or enzymatic assays. HPLC offers high accuracy but requires specialized equipment and trained personnel. First, urine samples are collected from patients over a 24 h period and kept refrigerated at 4 °C without any preservatives added. Prior to the analysis, the urine samples are acidified to a pH below 2 using hydrochloric acid. This acidification step is crucial, as it significantly improves the solubility of oxalates and affects the chromatography results. The HPLC method utilizes different setups across laboratories, with variations in columns, eluants, detectors, and retention times. The upper limits of linearity for HPLC-based methods vary between laboratories, ranging from 18 mg/L to 180 mg/L. It is worth noting that the HPLC method shows more variability in results compared to those obtained using the oxalate oxidase kit method, highlighting the importance of standardization and regular external validation to ensure accuracy and consistency in oxalate measurements across different laboratories. Enzymatic assays are more widely available and easier to perform but may be less accurate for very low or very high oxalate concentrations [90,91].

Urinary uric acid is typically measured in a 24 h urine collection using enzymatic methods or HPLC. The enzymatic colorimetric assay utilizes the uricase-catalyzed oxidation from uric acid to allantoin and H_2_O_2_ [92]. The generated H_2_O_2_ is then measured spectrophotometrically using 3-methyl-benzothiazoline-2-one hydrazone (MBTH) and 3-dimethylaminobenzoic acid (DMAB) under the catalytic influence of peroxidase. This reaction produces an intense blue indamine chromogen. The method is simple, sensitive, and selective, allowing for the rapid analysis of patients’ serum samples without deproteinization. It requires only 100 μL of serum for the colorimetric assay. The protocol for the HPLC detection of uric acid begins with diluting urine samples and acidifying them to a low pH to precipitate proteins [93]. The mixture is then filtered. Separation is achieved using a C-18 column. Detection is performed using a UV detector set at 235 nm. This wavelength is chosen based on the UV absorption spectrum of uric acid. Point-of-care devices for rapid uric acid measurement are also available, though they may be less accurate than laboratory-based methods.

Although these methods provide valuable information, they do have limitations. Twenty-four-hour urine collections can be inconvenient for patients and are subject to collection errors. Spot urine tests are more convenient but may not accurately reflect daily variations in metabolite excretion. Blood tests for some markers (e.g., oxalate) may not accurately reflect urinary excretion. Additionally, the need for specialized equipment and trained personnel for some of these tests can limit their availability in some clinical settings. Furthermore, these methods typically provide snapshot measurements rather than continuous monitoring, which may miss important temporal variations in metabolite levels. There is also a need for more rapid, point-of-care testing options for some of these biomarkers to facilitate timely diagnosis and treatment decisions. Advances in biosensor technology and point-of-care diagnostics [94,95,96] may offer promising avenues for addressing these limitations in the future.

## 3. Electrochemical Sensors in Nephrolithiasis Detection Research

### 3.1. Oxalate and Uric Acid Detections

In recent years, significant advancements have been made in the development of electrochemical sensors for the detection of key markers associated with nephrolithiasis. This section highlights the key research findings, focusing on the performances of different sensors in detecting oxalate and uric acid.

#### 3.1.1. Oxalate Detection

This subsection provides a comprehensive review of the mechanism of oxalate electrooxidation, strategies for the design of nanocatalytic materials, and advances in pH-dependent modulation. These aspects offer valuable theoretical support for the development of efficient and stable clinical sensing technologies. Oxalate (C_2_O_4_^2−^) is commonly present as a metabolic byproduct in human urine and plays a central role in forming the most prevalent type of kidney stones: calcium oxalate [76]. The electrochemical detection of oxalate typically relies on its oxidation at the electrode surface, generating measurable signals that correlate with the oxalate concentration [97]. Early studies reported that oxalate undergoes a two-electron transfer process, yielding carbon dioxide (CO_2_) as the final oxidation product, as shown in Equation (1):

(1) $$C_{2}O_{4}^{2-}\overset{-2e^{-}}{\to }2CO_{2}$$

Although the end product is thermodynamically stable CO_2_, the reaction proceeds via intermediate radical species. In particular, certain surfaces or electrode modifiers catalyze the formation of C_2_O_4_^−^ radicals at lower overpotentials, leading to increased current responses and enhanced sensitivity.

Because oxalate has a relatively high oxidation potential on bare electrodes (e.g., from +1.3 to +1.4 V vs. SCE on glassy carbon), sensor development frequently focuses on lowering the oxidation overpotential and enhancing electron transfer kinetics. Modified electrodes employing nanomaterials can provide active catalytic sites that bind and oxidize oxalate more easily than traditional surfaces. For instance, platinum group metals [98], WC nanoparticles, and palladium nano-shapes [99] on rGO have all been reported to reduce overpotentials and enhance peak currents. Platinum (Pt) and palladium (Pd) nanoparticles can serve as potent catalysts by lowering the activation energy of oxalate oxidation. Maiyalagan et al. [100] demonstrated that platinum nanoparticles supported on tungsten carbide nanotubes significantly decreased the oxidation overpotential for oxalate to ~220 mV lower than that of unmodified platinum. This synergy arises because WC itself exhibits “platinum-like” behavior, augmenting the catalytic effect of the Pt. In other approaches, silver nanorods on graphene [101] or recycled poly(lactic acid) composite electrodes [102] provide high surface areas, rapid electron transport, and adequate binding sites. These carbon-based materials promote the diffusion of oxalate to catalytic sites while reducing electrode fouling. The net result is an oxidation peak at lower potentials and more stable current responses.

Oxalate electrooxidation on modified electrodes generally involves two main steps. Oxalate anions diffuse from the bulk solution to the electrode surface. Specific functional groups or catalytic metal [103] sites can bind oxalate and facilitate the initial electron transfer. Oxalate is oxidized in a stepwise mechanism. In one frequently cited model, the first electron transfer yields a radical (C_2_O_4_^•−^), which can dissociate into CO_2_ plus CO_2_^•−^. The second electron transfer oxidizes CO_2_^•−^ to CO_2_. Ultimately, two electrons are consumed per C_2_O_4_^2−^ anion. On certain electrodes [104], the reaction may proceed more directly if the surface provides a strong catalytic effect, creating a concerted two-electron oxidation. The real pathway depends on the electrode material, pH, and local interactions at the interface.

Oxalate speciation is strongly pH dependent because oxalic acid has two acidic protons (p*K*_a1_ ≈ 1.25 and p*K*_a2_ ≈ 4.20 [105]). Below pH ~1.25, most oxalate is present as H_2_C_2_O_4_; between pH 1.25 and 4.20, HC_2_O_4_^−^ is encountered. Above pH ~4.2, oxalate is predominantly found in the form of C_2_O_4_^2−^. Because the ionic form can govern the adsorption affinity and oxidation kinetics, the peak potential and peak current for oxalate oxidation vary with pH. Basal plane pyrolytic graphite (BPPG) electrodes, for example, show about a 200–300 mV shift in the peak potential when shifting pH from 1 to 6 [97].

**Figure 5:**
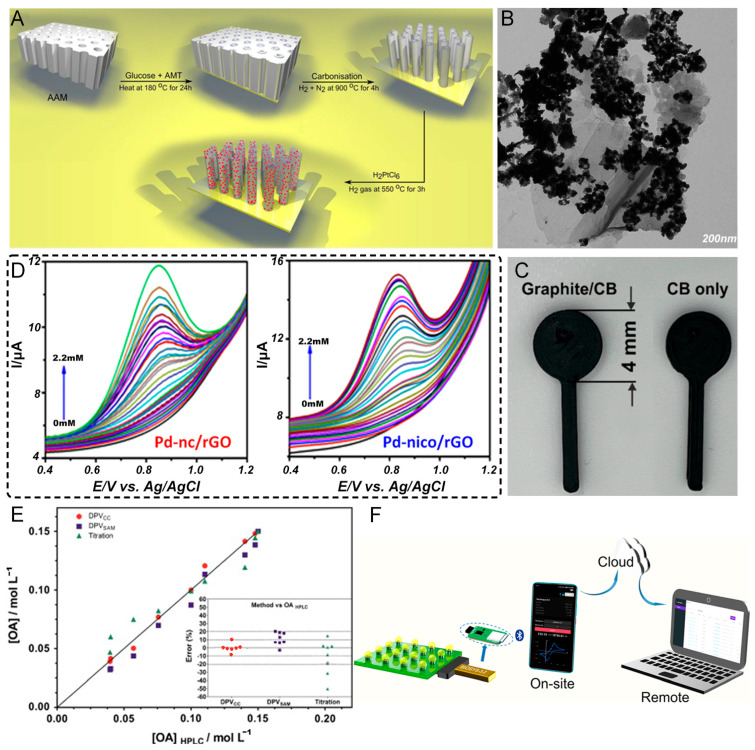
(**A**) The corresponding schematic representation of PtNPs loaded on the surface of WC NTs [100]. (**B**) The HR-TEM images of Gr with Ag NPs [101]. (**C**) Images of the lollipop AMEs printed from the bespoke graphite/CB and the CB/only filament [102]. (**D**) DPV measurements for Pd-nc/rGO-modified GCE and Pd-nico/rGO-modified GCE [106]. (**E**) Comparison of measurements performed using DPV with respect to expected HPLC results. Inset: the statistical error, mean, and standard deviation for each technique [107]. (**F**) Schematic diagram of a wireless-USB-like electrochemical platform for individual electrochemical sensing in microdroplets [108].

Oxalate is a major constituent of calcium oxalate stones, the most common type of kidney stones. Several innovative electrochemical sensing strategies have been developed for oxalate detection, each employing different electrode materials and modification approaches to achieve sensitive and selective detection. Maiyalagan et al. [100] developed a novel sensing platform based on platinum nanoparticles supported on tungsten carbide nanotubes (WC NT/PtNPs, Figure 5A), where the WC NTs with a large surface area (339 m^2^/g) serve as an ideal support for anchoring PtNPs uniformly, exhibiting excellent electrocatalytic activity toward oxalic acid oxidation, with a large decrease in the oxidation overpotential (220 mV) compared to those of unmodified and commercial Pt/C electrodes. Their sensor achieved high sensitivity (80 nA/nM) with a wide linear range (0–125 nM) and a low detection limit of 12 nM, with the enhanced performance attributed to the synergistic effect between the PtNPs and the platinum-like behavior of the WC. In contrast, Nagarajan and Sundramoorthy [101] reported a novel approach using a silver nanorod/graphene nanocomposite synthesized using 4-sulfocalixarene (SCX) as a soft template, where their one-pot electrosynthesis produced silver nanorods (~700 nm in length and ~60 nm in diameter) uniformly distributed on graphene sheets (Figure 5B), showing good electrocatalytic activity with a linear detection range of 3–30 mM and a detection limit of 0.04 mM while having higher detection limits than the WC NT/PtNP sensor. Their approach demonstrated good selectivity against common interferents and successfully detected oxalic acid in tap water samples.

Taking a different approach focused on sustainability, Arantes et al. [102] developed a mixed graphite/carbon black/recycled poly(lactic acid) (PLA) conductive filament for the additive manufacturing of electrodes (Figure 5C), with their graphite/carbon black composite showing excellent electrochemical performance (a heterogeneous rate constant of 1.26 × 10^−3^ cm/s) and achieving sensitive oxalate detection (in the linear range 10–500 μM, with a sensitivity of 0.0196 μA/μM and an LOD of 5.7 μM) while offering significant cost advantages by replacing 40% of the expensive carbon black with graphite. Exploring the impact of the nanoparticle morphology, Kesavan et al. [106] investigated palladium nano-shapes (nanocubes and nanoicosahedrons) supported on reduced graphene oxide (rGO), where Pd-nico/rGO showed superior performance compared to that of Pd-nc/rGO because of more exposed active sites from the icosahedral geometry, highlighting how nanoparticle shapes can significantly impact the sensing performance through different exposed facets (Figure 5D). Perhaps most notably, Hussain et al. [109] achieved ultra-sensitive detection using a molecular imprinting approach with tungsten carbide (W_2_C) nanoparticles and dopamine self-polymerization, where the molecularly imprinted polymer (MIP) layer created specific recognition sites for oxalic acid on the W_2_C-modified electrode. This sensor achieved remarkable sensitivity, with a wide linear range (0.1 nM–100 μM) and an extremely low detection limit of 0.04 nM, maintaining 94% of the initial activity after 35 days and successfully detecting oxalic acid in urine samples. The excellent selectivity was attributed to the specific imprinted cavities, while the W_2_C nanoparticles provided good conductivity and catalytic activity. When comparing these approaches, the MIP-W_2_C sensor achieved the lowest detection limit (0.04 nM), followed by WC NT/PtNPs (12 nM), while the silver nanorod/graphene and 3D-printed electrodes showed higher detection limits in the micromolar range, demonstrating how combining specific recognition sites with catalytic nanomaterials can achieve superior sensitivity. From a practical perspective, the 3D-printed electrode offers significant advantages in cost and sustainability through using recycled materials, while the silver- and platinum-based sensors likely have higher material costs, though the WC-based approaches provide more cost-effective alternatives to precious metals. In terms of detection methods, the difference between OA concentrations determined using DPV (Figure 5E) and HPLC lies below 10%. The method is less expensive than commonly used chromatography and other instrumental methods involving more toxic or expensive reagents. All the sensors demonstrated good stability and reproducibility, with the 3D-printed approach offering advantages in rapid prototyping and customization, while the MIP sensor showed excellent long-term stability, though the silver nanorod/graphene sensor required more careful control of the synthesis conditions. Importantly, most sensors successfully detected oxalate in real or synthetic urine/water samples, demonstrating practical applicability, with the MIP-W_2_C and TGM [110] sensors showing particularly good recoveries in complex matrices. Fluorescence sensors [99] are characterized by enhanced selectivity, when analyzing complex samples, as a result of their specialized probes. However, the high expense and vulnerability to environmental factors limit their suitability for accurate laboratory analysis. These diverse approaches showcase different strategies to achieve sensitive oxalate detection—from noble metal catalysts and carbon composites to molecular imprinting and additive manufacturing—each offering unique advantages in terms of sensitivity, cost, or practicality. The utilization of electrochemistry as a means of sensing (Figure 5F) provides options for different application requirements in clinical diagnostics and for monitoring kidney stone formation.

Table 1 provides a comparative overview of various electrochemical platforms for oxalate detection, highlighting their linear detection ranges, LODs, and real sample applications. By examining these data, several trends emerge. First, sensors employing noble metal nanoparticles (e.g., Au, Pt, and Pd) often demonstrate superior catalytic activities and lower detection limits, as observed with Pt NP@WC nanotubes [100] (LOD = 0.012 μM) and Pd NP@molecular sieve [111] (LOD = 0.40 μM). These materials benefit from high catalytic efficiency yet may be associated with the higher costs and limited availability of precious metals. Carbon nanomaterials, such as CNTs and rGO, offer large surface areas and robust electron transfer properties, which can be further enhanced by metal doping or functionalization. For example, Pt NP@rGO [112] and rGO/ionic liquid [113] sensors exhibit low detection limits (10 and 0.48 μM, respectively), primarily because of synergistic effects between the carbon matrix and metal additives or ionic liquids, improving both the catalytic activity and sensor stability. Moreover, the n-doping of rGO, as seen in Ag NP@n-doped rGO [114], can further optimize the charge transfer, contributing to an improved LOD of 2 μM. Another notable development is the use of ionic liquids and hybrid nanocomposites, such as TiO_2_–Fe NP/ionic liquid [113] and rGO/ionic liquid [115]. These materials offer improved conductivity and favorable electrocatalytic properties, translating to moderate LODs (23 and 0.48 μM, respectively) while maintaining good reproducibility and biocompatibility. In addition, the integration of molecular sieves, as in Pd NP@molecular sieve [111], demonstrates the potential for specific pore confinement to augment sensitivity toward oxalate. In addition, the table underscores the practical applicability of these sensors by highlighting their validation with real samples, such as urine, spinach, tomato, and onion. This confirms the feasibility of these sensors for food and clinical diagnostics. Overall, these sensors showcase remarkable versatility, sensitivity, and selectivity, with each approach offering distinct advantages—be it an ultra-low LOD, cost effectiveness, or easy fabrication—catering to diverse application scenarios in nephrolithiasis monitoring and broader oxalate detection needs.

#### 3.1.2. Uric Acid Detection

This section provides a comprehensive review of the mechanism of oxalate electrooxidation, strategies for the design of nanocatalytic materials, and advances in pH-dependent modulation. These aspects offer valuable theoretical support for the development of efficient and stable clinical sensing technologies. Uric acid is another key biomarker associated with kidney stone formation (uric acid stones), gout, and other metabolic disorders. In electrochemical sensing [122], uric acid is typically oxidized [123] at the electrode surface in a two-step, two-electron process to yield allantoin, CO_2_, and other byproducts. The classical mechanism for uric acid oxidation (Equation (2)) proceeds via an enol-type intermediate, which then undergoes further electron transfer and hydrolysis to ultimately produce allantoin as follows:

(2) $$C_{5}H_{4}N_{4}O_{3}+2H_{2}O\overset{2e^{-}}{\to }C_{4}H_{6}N_{4}O_{3}+2H^{+}+CO_{2}$$

The oxidation peak for uric acid often occurs in the range from +0.3 to +0.6 V vs. Ag/AgCl on various unmodified carbon electrodes. However, real samples pose multiple challenges because species such as ascorbic acid or dopamine can oxidize at similar potentials [124]. Therefore, designing advanced electrode surfaces that either shift the uric acid oxidation potential or selectively bind uric acid is a central theme in sensor research.

Like oxalate detection, uric acid detection greatly benefits from nanomaterials that accelerate electron transfer, enhance adsorption, or reduce oxidation overpotentials. However, the chemical structure of uric acid involves fused rings and nitrogen functionalities, which can lead to complicated reaction steps. The initial oxidation often produces a radical cation that either decomposes or couples with other species in solution. For a stable sensor response, electron transfer must occur rapidly, and the electrode surface should minimize side reactions.

Uric acid electrochemical biosensors can be divided into non-enzymatic [125] and enzymatic [126] variants. Non-enzymatic uric acid sensors rely on the direct oxidation of uric acid on the electrode. The mechanism typically proceeds by the formation of a radical cation (UA^•+^), followed by further electron transfer steps to produce allantoin. Interference from ascorbate and dopamine is a major concern, so the electrode material must suppress unwanted reactions or separate the redox peaks well. Enzymatic uric acid sensors use uricase to convert uric acid to allantoin and hydrogen peroxide (H_2_O_2_). The electrochemical signal typically tracks H_2_O_2_ rather than uric acid directly. Enzymatic approaches exhibit high specificity but can face stability issues because uricase is sensitive to pH, temperature, and potential enzyme denaturation over time.

Uric acid’s ionization state is affected by the pH (p*K*_a_~5.75). Below this p*K*_a_, uric acid is predominantly uncharged; above it, the deprotonated form (urate anion) becomes dominant [97]. This shift impacts how the molecule adsorbs on electrode surfaces and how easily it undergoes electron transfer. Many sensors operate in weakly acidic or neutral pH (e.g., phosphate-buffered solutions around pH 7) environments to mimic physiological fluids and to maintain stable enzymatic activities in enzymatic sensors [127]. For non-enzymatic UA oxidation, certain metal oxides or polymer coatings are also pH dependent. Adjusting the electrolyte composition, ionic strength, and buffer capacity helps to fine tune the sensor’s performance and limit matrix effects in real samples (e.g., urine, saliva, and serum). In enzymatic sensors [128], uricase offers intrinsic selectivity for UA. However, the resulting H_2_O_2_ can still be oxidized at potentials where interfering species also appear. Some sensors incorporate additional protective layers, such as Prussian blue or polymer membranes, to reduce cross-reactivity.

UA and its oxidation products can foul the electrode, causing diminished responses over repeated measurements. Nanomaterials with large surface areas and robust chemical stability—like carbon nanotubes [129] or platinum group metals [130]—are less prone to passivation. In enzymatic designs, the entrapment of uricase in polymeric matrices or cross-linked hydrogels [131] can protect the enzyme from deactivation while retaining access to substrate molecules. Nonetheless, long-term stability remains a challenge, especially in point-of-care testing.

**Figure 6:**
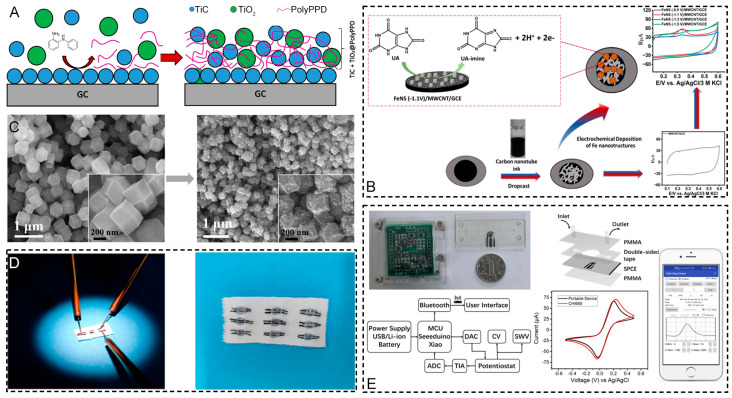
(**A**) Schematic diagram of the growth of PolyPPD and the incorporation of nanoparticles within the polymer matrix [132]. (**B**) A schematic diagram showcasing the development of an iron (Fe)-nanostructured (FeNS) sensor for the electrochemical detection of UA and the mechanism of the electrooxidation process of uric acid [133]. (**C**) SEM images of synthetic BMZIF and its resultant CNCo [134]. (**D**) Photographs of FECTs integrated into the fabric and the UA sensor during testing [135]. (**E**) Portable-device fabrication [136].

Uric acid is a key risk factor for the formation of uric acid stones and can also contribute to the development of calcium oxalate stones. Various electrochemical sensing approaches have been developed for the sensitive and selective detection of uric acid. The principal strategies include the direct oxidation of uric acid on modified electrodes and integration with advanced sensing platforms. Zenasni et al. developed [132] a composite material based on titanium carbide (TiC) and TiO_2_ entrapped in a poly(N-phenyl-o-phenylenediamine) (PolyPPD) polymer matrix through electrochemical synthesis. The TiC and TiO_2_ nanoparticles enhanced the conductivity and electron transfer kinetics, while the polymer matrix provided selectivity through specific binding sites (Figure 6A). This sensor achieved selective UA detection without interference from ascorbic acid, with a linear range of 2–110 μM and detection limit of 0.83 μM. Taking a different approach, Joshi and Slaughter fabricated [133] multiwalled carbon-nanotube-supported Fe-nanostructured interfaces through electroreduction. The synergistic combination of carbon nanotubes and iron nanostructures (100–120 nm) provided abundant active sites and accelerated the electron transfer (Figure 6B), enabling sensitive uric acid detection from 5–500 μM with a detection limit of 3.26 μM. Liu et al. [134] explored a novel strategy using a bimetallic metal–organic framework (BMZIF)-derived porous carbon as the sensing material. The large surface area and well-distributed active sites of the N,Co-doped porous carbon (Figure 6C) enhanced the electrocatalytic activity, achieving UA detection from 2 to 110 μM with a 0.83 μM detection limit. For wearable applications, Tao et al. [135] developed a fiber-based organic electrochemical transistor integrated with a molecularly imprinted polymer membrane. The PEDOT nanocluster structure on rGO-modified cotton fibers provided high conductivity, while the molecular imprinting enhanced the selectivity, enabling UA detection from 1 nM to 500 μM (Figure 6D). From a different perspective, Zhang et al. [136] focused on the sensing platform by developing a portable microfluidic electrochemical sensor with a novel nonlinear fitting strategy (Figure 6E). The integration of microfluidics improved the stability, with an RSD of <1%, while the nonlinear model expanded the detection range to 5–1000 μM using unmodified screen-printed carbon electrodes. Comparing these approaches reveals different strengths—the TiC/TiO_2_/PolyPPD composite and BMZIF-derived carbon excel in selectivity and moderate sensitivity; the Fe/CNT interface balances good sensitivity with a practical detection range, and the fiber-based transistor enables wearable sensing with ultra-high sensitivity, while the microfluidic platform with the nonlinear model achieves the widest detection range. The detection limits range from nanomolar to micromolar levels, with linear ranges spanning 2–3 orders of magnitude, except for the microfluidic system’s expanded range through nonlinear modeling. Common strategies for enhancing the performance include (1) using nanostructured materials to increase active sites and accelerate the electron transfer; (2) incorporating selective recognition elements, like molecular imprinting or polymer matrices; (3) optimizing the sensing platform through transistor-based amplification or microfluidic integration; and (4) developing advanced data analysis models. The choice of the approach depends on the specific application requirements of the fiber-based transistor suits for wearable monitoring [135,137]; the microfluidic platform enables point-of-care testing [138], while the nanostructured materials provide reliable benchtop sensing [139,140,141,142].

Most systems demonstrated good selectivity against common interferents, like ascorbic acid, glucose, and dopamine, though the levels of the selectivity varied. The microfluidic approach showed excellent stability and reproducibility because of the continuous flow, while the other systems exhibited varying degrees of signal drift over repeated measurements. Looking at practical aspects, the screen-printed and fiber-based platforms offer better potential for commercialization because of their simpler fabrication, while the nanostructured materials may face scaling challenges despite their superior performances. Cost considerations also favor simpler platforms over complex nanostructured materials. Table 2 highlights a wide range of electrode configurations and nanomaterial modifications for uric acid detection, reflecting diverse strategies to balance sensitivity, selectivity, and real-world applicability. Several observations emerge from examining the reported LOD and linear ranges. First, sensors [103,143] based on complex metal/metal oxide frameworks (e.g., SN-ZIF/CF [140] and COF-NH_2_-MWCNT-Au [144]) often exhibit exceptionally low LODs (as low as 0.0056 μM), underscoring the roles of porous architectures and large surface areas in increasing active sites and enhancing the electron transfer. Similarly, the combination of MWCNTs with doped or functionalized materials (e.g., CoPc-MWCNT [145] and Co-N/C@MWCNT [146]) offers broad linear ranges and improved conductivity, although detection limits can vary considerably. Another intriguing trend is the integration of molecularly imprinted polymers or polymeric films, such as MIP/PEDOT/carbon fiber [135] or Poly(DPA)/SiO_2_@Fe_3_O_4_/CPE [147]. These platforms leverage specific binding cavities or polymer-mediated interactions to enhance selectivity, achieving ultra-low LODs (0.001 μM) while successfully detecting uric acid in simulated or real biological fluids. Moreover, the development of flexible and wearable sensors, exemplified by PyTS@Ti_3_C_2_T_x_ for sweat analysis [137], highlights the growing emphasis on noninvasive continuous monitoring in personal healthcare. In terms of practical applications, the majority of the sensors (e.g., GO-Silane [148] and He@CNT/alk-Ti_3_C_2_T_x_/CHIP [138]) have been validated with clinically relevant matrices, like urine, serum, or artificial urine, indicating strong translational potential for point-of-care testing. Notably, some sensors achieve a compromise between high sensitivity and a wide detection range (e.g., PAMAM/MWCNT-AgNP/PNR [139], measuring from nanomolar to millimolar levels), which is essential for monitoring fluctuating uric acid concentrations in diverse patient populations.

## 4. Current Challenges and Barriers to Clinical Application

Electrochemical sensors face several challenges in the clinical translation of kidney stone detection. At a technical level, the difference between the concentrations of highly concentrated background substances (e.g., urea, creatinine, and proteins) and stone markers (e.g., calcium oxalate nanocrystals and uric acid microcrystals) in urine can be up to 6–8 orders of magnitude, resulting in an insufficient signal-to-noise ratio of the sensor. In addition, similar electrochemically active interferents (e.g., ascorbic acid and dopamine) can cause fake signals. High-surface-area/catalytically active materials (e.g., nitrogen-doped graphene) are used to enhance the responses of targets, or the molecular sieve effect of metal–organic frameworks (MOFs) is exploited to selectively trap small-molecule markers. Different stone types (e.g., calcium based and uric acid) require the detection of Ca^2+^, C_2_O_4_^2−^, and uric acid, but multichannel sensors are prone to the cross-reactivity or crosstalk of signals, reducing the detection accuracy. Overlapping signals are resolved by integrating electrode arrays with different modification materials (e.g., C_2_O_4_^2−^-selective ion carriers and uric-acid-oxidase-modified electrodes).

For clinical validation and standardization, fluctuations in urine pH (4.5–8.0) have been observed to compromise the stability of ionic markers. Additionally, the presence of cellular debris in turbid samples potentially obstructs microfluidic channels or contaminates electrode surfaces. The development of an integrated microfluidic device that combines a contamination-resistant electrode with centrifugation and filtration is a potential solution to this problem. This device would enable ‘sample-in-result-out’ testing and the creation of an artificial urinary reference material with a known concentration of markers for device calibration. In addition to this, stone formation is characterized by its dynamism (e.g., intermittent hyperoxaluria) and existing clinical standards (e.g., 24 h urine biochemistry), which are not commensurate with the timeliness of the sensor’s immediate detection, resulting in biased validation data. The development of a wearable sensor for the continuous collection of urine during both daytime and nighttime hours, in conjunction with temporal analysis to identify fluctuating patterns of risk of stone formation, is therefore recommended.

In practical terms, the use of precious metal electrodes and complex nanomaterials is costly, and existing processes are difficult to meet the homogeneity requirements for large-scale production, with deviations of >15% in the electrode modification layer’s thickness being a common occurrence. The development of robust composite or carbon-based electrodes, optimized for low-cost printing and minimal environmental impact, could significantly expand the global reach of these technologies.

## 5. Conclusions

Electrochemical sensors have demonstrated significant promise in the early detection and monitoring of nephrolithiasis, offering substantial advantages over conventional diagnostic methods in terms of sensitivity, real-time capability, and potential for point-of-care use. Through a critical examination of the latest research, it is clear that advances in electrode materials—ranging from precious metal nanoparticles to carbon-based composites—have markedly enhanced the sensitivity and selectivity of these platforms for key biomarkers, such as oxalate and uric acid. In particular, innovative designs employing molecular imprinting, nanozyme-based catalysis, and 3D printing methodologies underscore the versatility and adaptability of electrochemical approaches. Despite these encouraging developments, several overarching challenges continue to limit the widespread clinical translation of electrochemical sensors. Moving forward, integrating electrochemical sensors with a digital health infrastructure represents a key next step. Cloud-based data analysis and telemedicine platforms could facilitate remote monitoring, allowing clinicians to track fluctuations in biomarkers and tailor treatments proactively. In sum, although many challenges lie ahead, the continued evolution of electrochemical sensors has the potential to revolutionize nephrolithiasis diagnostics, improve treatment precision, and, ultimately, reduce the significant health and economic burdens of kidney stone disease.

## Figures and Tables

**Table 1:** Sensing performances of recent electrochemical sensors for oxalate detection.

**Sensor**	**Linear Detection Range (μM)**	**Limit of Detection (μM)**	**Real Sample**	**Ref.**
Pd nanocube@rGO	49.5–10,000	50		[106]
Ag nanorod@rGO	3000–30,000	40	tap water	[101]
CuS nanospheres	50–700	35.6		[116]
TiO_2_–Fe NP/ionic liquid	500–3000	23	urine	[113]
Au NP–polypyrrole@rGO	50–7000	20		[117]
Pd NP@functional CNTs	30–5000	20	spinach	[118]
CNTs	50–150	12	spinach	[119]
Pt NP@rGO	100–50,000	10	spinach	[112]
TGM organic fluorescent probe	0–0.16	3.86	vegetable	[110]
Graphite@Ag–AgCl	10–750	3.7	urine	[120]
Ag NP@n-doped rGO	10–300	2		[114]
Pt–Pd NP@n-doped rGO	1.5–500	0.84		[121]
N-CD-MnO_2_ NSs	1–50	0.69	urine	[99]
rGO/ionic liquid	8–6000	0.48	spinach	[115]
Pd NP@molecular sieve (SBA-15)	10–140	0.40	onion and tomato	[111]
Pt NP@WC nanotubes	0.012–0.125	0.012	tomato	[100]

**Table 2:** Sensing performances of recent electrochemical sensors for uric acid detection.

**Sensor**	**Linear Detection Range (μM)**	**Limit of Detection (μM)**	**Real Sample**	**Ref.**
CoPc-MWCNT	125–4000	260	urine	[145]
ZnGa_2_O_4_: Mn^2+^@MnO_2_ fluorescent	20–50	1.3	serum	[149]
CNCo	2–110	0.83	serum	[134]
N-doped SiC	3.7–125	0.77	urine	[150]
PyTS@Ti_3_C_2_T_x_	5–100	0.48	sweat	[137]
He@CNT/alk-Ti_3_C_2_T_x_/CHIP	1–1000	0.41	urine	[138]
Poly(DPA)/SiO_2_@Fe_3_O_4_/CPE	1.2–8.2	0.4	urine	[147]
COF-NH_2_-MWCNT-Au	0.3–200	0.29	urine	[145]
ZrO_2_/ZnO	10–2400	0.29	serum	[143]
Methylene blue-cPDA/graphene paper	0.6–350	0.2	serum	[151]
CoV/MWCNT-COOH	1–100	0.1	urine	[152]
Co-N/C@ MWCNT	1–40	0.09	serum	[146]
Ti-C-Tx	0.5–4; 100–1500	0.075	urine	[153]
GO-Silane	2.5–80	0.065	urine	[148]
n-HA/CPE	0.068–50	0.05	urine	[141]
SN-ZIF/CF	0.01–2800	0.0056	serum	[140]
PAMAM/MWCNT-AgNP/PNR	0.016–2500	0.005	urine	[139]
MIP/PEDOT/carbon fiber	0.001–500	0.001	artificial urine	[135]

## Data Availability

This study is a review article and does not involve original data. All cited data sources are listed in the references.
